# Adsorptive removal of malachite green dye from aqueous solution using *Rumex abyssinicus* derived activated carbon

**DOI:** 10.1038/s41598-023-41957-x

**Published:** 2023-09-07

**Authors:** Mikiyas Abewaa, Ashagrie Mengistu, Temesgen Takele, Jemal Fito, Thabo Nkambule

**Affiliations:** 1 Department of Chemical Engineering, College of Engineering and Technology, Wachemo University, P. O. Box 667, Hossana, Ethiopia; 2 The Federal Democratic Republic of Ethiopia, Manufacturing Industry Development Institute, P. O. Box 1180, Addis Ababa, Ethiopia; 3 Institute for Nanotechnology and Water Sustainability (iNanoWS), College of Science, Engineering and Technology, University of South Africa, Florida Science Campus, Johannesburg, 1710 South Africa

**Keywords:** Environmental sciences, Engineering

## Abstract

The potential for malachite green dye saturated effluent to severely affect the environment and human health has prompted the search for effective treatment technologies. Thus, this study was conducted with the goal of developing activated carbon from *Rumex abyssinicus* for the adsorptive removal of malachite green dye from an aqueous solution. Unit operations such as drying, size reduction, impregnation with H_3_PO_4_, and thermal activation were used during the preparation of the activated carbon. An experiment was designed considering four main variables at their respective three levels: initial dye concentration (50, 100, and 150 mg/L), pH (3, 6, and 9), contact period (20, 40, and 60 min), and adsorbent dosage (0.05, 0.01, and 0.15 g/100 mL). Optimization of the batch adsorption process was carried out using the Response Surface methodology's Box Behnken approach. The characterization of the activated carbon was described by SEM for surface morphology with cracks and highly porous morphology, FTIR for multi-functional groups O–H at 3506.74 cm^−1^ and 3290.70 cm^−1^, carbonyl group stretching from aldehyde and ketone (1900–1700 cm^−1^), stretching motion of aromatic ring C=C (1543.12 cm^−1^), stretching motion of –C–H (1500–1200 cm^−1^), vibrational and stretching motion of –OH (1250.79 cm^−1^), and vibrational motion of C–O–C (1049.32 cm^−1^), pHpzc of 5.1, BET for the specific surface area of 962.3 m^2^/g, and XRD for the presence of amorphous structure. The maximum and minimum dye removal efficiencies of 99.9% and 62.4% were observed at their respective experimental conditions of (100 mg/L, 0.10 mg/100 mL, pH 6, and 40 min) and (100 mg/L, 0.15 mg/100 mL, pH 3, and 20 min), respectively. Langmuir, Freundlich, Toth, and Koble-Corrigan models were used to evaluate the experimental data, in which Koble-Corrigan model was found to be the best fit with the highest value of R^2^ 0.998. In addition to this, the kinetic studies were undertaken using pseudo-first-order, pseudo-second-order, intraparticle diffusion, and Boyd models, and as a result, the pseudo-second-order model proved to have a better fit among the kinetic models. The kinetics and isotherm analysis revealed that the nature of the adsorption to be homogenous and monolayer surfaces driven by chemosorption. Furthermore, the thermodynamics study revealed the nature of adsorption to be feasible, spontaneous, and endothermic. On the other hand, the reusability study depicted the fact that the adsorbent can be utilized for five cycles with a negligible drop in the removal efficiencies from 99.9 to 95.2%. Finally, the low-cost, environmentally benign, and high adsorption capacity of the adsorbent material derived from *Rumex abyssinicus* stem could be used to treat industrial effluents.

## Introduction

Uncontrolled industrialization and urbanization have been proven to be the major contributors to the depletion of natural resources as well as varying degrees of environmental degradation. In line with this, rapid industrialization, urbanization, and improper utilization of natural resources end up with the discharge of substantial amounts of untreated wastewater, which puts fresh water under stress^1,2^. Moreover, the current environmental condition has reached the Antrophocene epoch of the geological time scale, where human activities are highly responsible for the environmental pollution our planet is facing nowadays^3^. Hence, initiatives to ensure the sustainability of industrialization, both in terms of preventing environmental damage^4^ and fostering more environmentally friendly industries are urgently needed^5^. Different organic, microbiological, and inorganic contaminants are becoming the cause of most water pollution, posing major environmental and human health challenges. These pollutants are either manufactured or naturally occurring substances that enter the environment through anthropogenic activities, including freshwater sources and wastewater^6^. On the other hand, increased demand for textile products necessitates the expansion of textile industries, leading to a dramatic increase in wastewater discharge, making the sector one of the most serious sources of pollution worldwide^7^. Dyes, degradable organics, detergents, stabilizing agents, desirers, inorganic salts, and heavy metals are among the contaminants found in textile industry effluent^8^.

Malachite green (4[4-(dimethylamino) phenyl] (phenyl) methylidene-N N-dimethylcyclohexa-2, 5-dien-1-iminium chloride) is a solid, cationic, and crystalline dye characterized by a molecular formula of C_23_H_25_CIN_2_ and a molecular weight of 346.11 g/mol^9,10^. Additionally, its higher solubility in water, synthetic nature, higher stability, and affordability increased malachite green demand in various industrial sectors^11,12^. It is widely applied in manufacturing industries like textile dying, leather, food, pulp and paper production, paint and pigment production, wool, and silk^11^. Moreover, malachite green is used as an antiseptic, parasiticide, and fungicide in aquaculture^13^. However, there are environmental and human health issues associated with malachite green dye. Malachite green is persistent (resists natural bio-degradation), bio-accumulative, mutagenic, carcinogenic, and teratogenic^14–16^. Furthermore, malachite green ingested through water poises the kidney, heart, and breast, which are vital organs of humans^10^. Diseases such as skin, gastrointestinal, and respiratory irritation are associated with the ingestion of malachite-contaminated water^15^. Moreover, the biochemical reduction of malachite green in the mammalian body system forms a chemical substance that, if accrued in the body for more than five months, increases liver tumors and facilitates lung adenomas in F344 males known as leucomalachite (LMG)^9^. The reduction of water surface oxygenation resulting from the obstruction of light penetration caused by the discharge of malachite green dye effluent puts aquatic life in peril^17^. Indeed, the use of malachite green as a food coloring additive is banned in countries like the USA, Canada, China, the UK, and the European Union^18^. Hence, for environmental preservation and human health protection, the decontamination of persistent dyes like malachite green has become a serious agenda item for the global community.

Due to the ineffectiveness of conventional wastewater treatment techniques for decontamination of persistent and toxic pollutants like malachite green dye, advanced wastewater treatment methods have become the choice of experts working in the areas of public health and environmental protection ^19^. Scholars have extensively researched the decolorization of malachite green dye using advanced wastewater technologies. For instance, membrane filtration ^20,21^, Fenton oxidation ^22^, ultraviolet assisted advanced oxidation ^23,24^, and catalyst assisted advanced oxidation process ^25^. However, these technologies have their own limitations, such as the lack of sustainability and short life span of membrane technology due to the clogging of filter pores by dye molecules and the exaggerated cost to acquire^26,27^, capital intensiveness, tailoring of complex chemistry, excess peroxide requirement for advanced oxidation processes, requirement of large amounts of chemicals, and the generation of excessive toxic sludge by chemical precipitation^28^, unbearable investment cost and expensive electricity supply for electrochemical methods^29^. On the other hand, adsorption, being low-cost, eco-friendly, effective, sustainable, and non-complex, has become an alternative pollutant detoxification mechanism^30^. Activated carbons, biochar, and magnetic biochar/activated carbons are a class of adsorbents whose state of art is thoroughly established^31^. Commercial activated carbon, which is widely installed in various industrial wastewater treatment sectors, is known for its excellent adsorption capability and effectiveness^32^. Even though commercial activated carbon is efficient and reliable, the preparation cost and non-sustainability limit its application^33^. Hence, many researchers have continued to search for adsorbent materials that are especially versatile in terms of both local availability and low cost.

Nowadays, the development of biomass-based adsorbent materials has been thoroughly investigated to overcome the limitations associated with commercial activated carbons. To make sure this, the inexpensive, freely available, and easily utilizable agro-waste-based adsorbent materials^34^ such as banana peel ^35^, orange peel ^36^, watermelon peel ^37^, and desert date seed shell ^38^ are under investigation for their effectiveness in removing malachite green dye. However, these studies have the limitations of low adsorption capacity, a substantial amount of adsorbent consumption for a small amount of pollutant adsorption, a small specific surface area, and a long contact time for pollutant detoxification. Hence, *Rumex abyssinicus*, a plant that is mostly distributed in tropical Africa, specifically Ethiopia, has been selected to tackle disadvantages like low removal efficiency and the longer contact time requirement of biomass-based activated carbons. So far, the application of the roots and root barks of *Rumex abyssinicus* for medicinal purposes has been thoroughly studied^39–41^. However, a few studies^42,43^ have been conducted to valorize the stem of *Rumex abyssinicus* for environmental clean-up. Moreover, the previous studies did not consider the thermodynamics study, which has a great implication in the design of the adsorption system. Furthermore, the ratio of precursor material (*Rumex abyssinicus*) to activating agent (H_3_PO_4_) was 1:3 and 1:2, which is very high and results in a substantial amount of chemical consumption. On the other hand, linear forms of isotherm and kinetics models were used, resulting in errors in data analysis. Hence, this research work was conducted with the aim of filling the gaps mentioned above. So far, the valorization of the stem and leaves of *Rumex abyssinicus* for the removal of malachite green dye from aqueous solutions or industrial wastewater is missing. Therefore, this study was aimed at investigating the adsorptive performance of *Rumex abyssinicus*-derived activated carbon for the removal of malachite green dye from aqueous solution with the simultaneous evaluation of isotherm, kinetics, and thermodynamics studies and examining the regeneration and reusability potentials of the developed adsorbent.

## Materials and methods

### Adsorbent preparation

The precursor material used for activated carbon synthesis, i.e., the stem of *Rumex abyssinicus*, was collected from Addis Ababa, Ethiopia. The collected sample was thoroughly washed with tap water before being rinsed with distilled water. The dust-freed sample was sundried and then placed in the oven (Model BOV-T50F) at 105 °C for 24 h to remove the remaining moisture. Thereafter, the oven-dried sample was impregnated with phosphoric acid in a 1:1(m/v) ratio and soaked thoroughly. Then, it was oven-dried for 8 h after which it was pyrolyzed in the furnace at 500 °C for 2 h. The prepared adsorbent material was then taken out of the furnace, cooled in a desiccator, and thoroughly washed with distilled water until its pH became neutral. Finally, the activated carbon was ground to a particle size of 250 ${\upmu {\rm m}}$ and placed in airtight plastic until the subsequent experiments^44,45^. All the methods were carried out in accordance with relevant guidelines and legislation of institutional, national, and international.

### Adsorbent characterization

The Rumex abysiniccus-derived adsorbent was characterized for pH point of zero charge (pHpzc), scanning electron microscope (SEM), Brunner emitter and teller (BET), Fourier Transform Infrared (FTIR) spectroscopy, and X-ray diffraction (XRD) following methods described by^42^.The functional group present in the adsorbent material as well as its crystalline nature were determined using the Fourier transform infrared Shimazu IRAffinity-1 s and X-ray diffraction XRD (Rigabu Miniflex 600n diffractometer), respectively. The FTIR scanning was carried out at a wavelength range of 400–4000 cm^−1^. However, XRD scanning was performed along 2 of 10$^\circ$–80$^\circ$. Additionally, SEM and BET were used for surface morphology and specific surface area determination, respectively^46,47^.

### Malachite green adsorption experimental design

An experimental design considering four variables with their respective three levels was established to examine the use of activated carbon obtained from *Rumex abyssinicus* for the removal of malachite green from an aqueous solution. The response variable is removal efficiency (%), and the independent variables are pH, contact time, initial dye concentration, and adsorbent dosage. The levels of the experimental variables were denoted as lower (−), middle (0), and higher (+). In essence, 81 experimental runs were produced via 3^4^ factorial designs. However, to reduce the number of experimental runs to 30, the design expert's optimization strategy was utilized. Table 1 indicates the experimental design for the Box-Behnken technique approach to the response surface methodology. The experimental values are set in accordance with the results of earlier experiments.

**Table 1:** Box Behnken approach of experimental design.

Variables	Unit	Low (−)	Middle (0)	High (+)
pH		3	6	9
Adsorbent dosage	g/100 mL	0.05	0.1	0.15
Dye concentration	mg/L	50	100	150
Contact time	min	20	40	60

### Batch adsorption experiments for malachite green dye removal

The batch adsorption experiment for malachite green dye removal through *Rumex abyssinicus*-derived adsorbent was conducted in a 100-mL solution. A malachite green dye solution was prepared by dissolving 50, 100, and 150 mg into 1 L of distilled water separately. This gave a dye concentration of 50, 100, and 150 mg/L, respectively. The solution pH was adjusted using NaOH (0.1 M) and HCl (0.1 M) based on the required acidity and basicity of the solution. Then, a fixed amount of adsorbent dosage was added to an Erlenmeyer flask containing a 100-mL pH-adjusted solution of malachite green dye. Thereafter, the solution was stirred using an orbital shaker at 125 rpm for the specific duration of contact time. As contact time completed, the solution was filtered using Whatman filter paper 42, where supernatants were removed, and the filtrate was used for the determination of the amount of dye remaining in the aqueous solution. Then, various sampling bottles were used to collect the dye-containing supernatants, which in turn were used to determine the amount of dye remaining in the solution. The amounts of malachite green dye remaining in the solution were collected using various sampling bottles. Finally, the final dye concentration was determined using an ultraviolet (UV)-visible spectrophotometer (Agilent Technology, Cary 100 UV–visible Spectrophotometer) at a maximum wavelength of 620 nm. For calibration curve development, the stock solution for malachite green dye was prepared by weighing 1 g of malachite green powder and dissolving it into 1 L of solution to get 1000 mg/L of malachite green dye solution. Thereafter, a serial dilution was performed using distilled water in order to get the desired malachite green dye concentrations of 30, 50, 70, 90, 110, 130, and 150 mg/L. Thereafter, the standard solutions are transferred into cuvettes. The standard solution containing cuvettes is placed in the UV–VIS spectrophotometer at a maximum wavelength of 620 nm. Then, absorbance readings for each sample were obtained and recorded on a spread sheet. The absorbance data (y axis) was plotted against the concentration (x axis) and examined for the best linear curve fit. Finally, the unknown concentrations were determined using an equation generated from a linear plot. The removal efficiency and adsorption capacity were calculated using Eqs. (1) and (2), respectively.

1 $$\mathrm{R}\left({\%}\right)=(\frac{{\mathrm{C}}_{\rm{i}}-{\mathrm{C}}_{\rm{f}}}{{\mathrm{C}}_{\rm{i}}})*100.$$

2 $$\mathrm{Qe}=\left(\frac{\mathrm{Co}-\mathrm{Ce}}{\mathrm{m}}\right)*\mathrm{V}$$

where, R is removal efficiency, Qe is adsorption capacity, m is mass of the adsorbent, V is volume of the solution, Ci and Cf are initial and final malachite green concentration respectively^48^.

### Adsorption isotherm

Adsorption isotherms provide information regarding the interaction of adsorbate and adsorbent at equilibrium. Moreover, these equilibrium relationships help us understand the basic adsorption mechanisms and surface characteristics of adsorbate and adsorbent. There are various adsorption isotherms used in modelling the adsorption isotherm, of which Langmuir and Freundlich isotherm models are the most widely used ones in the adsorption system. The Langmuir isotherm model assumes a monolayer and homogenous adsorption surface with strong binding of adsorbate and adsorbent, whereas the surface of the adsorption system is expected to improve itself in the Freundlich isotherm. In the Freundlich isotherm model, there is heterogeneous and multilayer surface interaction. Moreover, the adsorption mechanism is said to be chemisorption if Langmuir isotherm was found to be explanatory and physical sorption when Freundlich isotherm was found to represent more of the adsorption data. The adsorption isotherm study was undertaken keeping pH, contact time, and adsorbent dosage at 6, 40 min, and 0.1 g/100 mL, respectively, while varying the initial dye concentration (50, 70, 90, 110, 130, and 150 mg/L). The Langmuir and Freundlich equations are presented in Eqs. (3) and (4), respectively.

3 $$\frac{QmaxKLCe}{1+KLCe}$$

4 $$ {\text{Qe}} = {\text{KFCe}}^{{{1}/{\text{n}}}} $$

Additionally, the adsorption dimensionless factor constant (RL) that is used to estimate Langmuir isothermal feasibility is indicated by Eq. (5).

5 $$\mathrm{RL}=\frac{1}{1+{\mathrm{K}}_{\rm{L}}\mathrm{Ce}}$$

where q_max_ is the maximum Langmuir adsorption capacity, K_L_ is the Langmuir constant related to adsorption capacity, and K_F_ and n are Freundlich constants related to adsorption capacity and intensity^49^. In addition, Toth and Koble-Corrigan isotherm models were investigated in order to further examine the best data fit. Equation (6) shows the relation between parameters for Toth isotherm models, where K and nt are Toth isotherm constants indicating surface affinity and degree of heterogeneity, respectively^50,51^.

6 $$\mathrm{Qe}= \frac{{Q}_{max}KCe}{[1+(K{Ce)}^{nt}){]}^{1/nt}}$$

Similarly, the Koble-Corrigan model is shown in Eq. (7), where parameters such as A and B are Koble-Corrigan constants in (mg/g) and (L/mg)^n^, respectively. Meanwhile, adsorption intensity is represented by nk^52,53^.

7 $$\mathrm{Qe}= \frac{A{{C}_{e}}^{nk}}{(1+B{{C}_{e}}^{nk})}$$

### Adsorption kinetics

Adsorption kinetics is studied to determine the potential rate-controlling step in the adsorption process. Moreover, that also allows for an understanding of kinetic mechanisms and the rate of dye uptake. In the current study, the four widely used kinetics models were examined to determine the nature of the adsorption that occurs between *Rumex abyssinicus*-derived activated carbon and malachite green dye. These kinetic models are pseudo-first-order (PFO), pseudo-second-order (PSO), Intraparticle diffusion and Boyd models. Normally, PFO is related to physical adsorption, whereas PSO is associated with chemisorption. Fundamentally, chemical sorption results in strong attachment between adsorbate and adsorbent with slow attachment. However, in physical adsorption, the adsorption process is fast with weak interactions between adsorbate and adsorbent. Hence, physical adsorption is easily reversible, whereas it is difficult to reverse chemical adsorption. pH 6, an adsorbent dosage of 0.1 g/100 mL, and an initial dye concentration of 100 mg/L with varying contact times of 20, 30, 40, 50, and 60 min were used for the kinetics study. Basic equations used to determine parameters related to PFO and PSO are presented in Eqs. (6) and (7), respectively.

8 $$ Qt = Qe(1 - e^{( - K1t)} ) $$

9 $$\mathrm{Qt}=\frac{{\mathrm{Qe}}^{2}{\mathrm{K}}_{2}\mathrm{t}}{1+{\mathrm{QeK}}_{2}\mathrm{t}}$$

where the ${q}_{t} \mathrm{and} {q}_{e}$ (mg/g) are adsorption capacity at time t and equilibrium adsorption capacity, respectively; ${K}_{f} \mathrm{and} {K}_{s}$ are pseudo-first-order (g/(mg min)) and pseudo- second-order (g/(mg min)) adsorption rate constants, respectively ^54^. Intraparticle diffusion and Boyd models are used to examine the presence of films or bulk diffusion in the adsorption process. If the intraparticle diffusion model is found to fit the data best, it is the bulk diffusion that governs the rate of adsorption. Equations (10) and (11) depict the relations between parameters for intraparticle diffusion and Boyd models, respectively.

10 $${\mathrm{Qe}=\mathrm{ Ki}t}^{1/2}+\mathrm{Ci}$$

11 $$\mathrm{F }= \frac{Qt}{Qe}=1- (\frac{6}{{\pi }^{2}})\mathrm{ exp }(-\mathrm{Bt})$$

where Ki is the intraparticle diffusion constant, Ci is concentration, F is the fraction of pollutant adsorbed at time t, and B_t_ is the mathematical function of F^55,56^.

The criteria for best fitting were evaluated using error analysis (reduced chi square) and the coefficient of determination (R^2^). The coefficient of determination represents the variance about the mean; it is used as a model performance indicator in adsorption kinetics and isotherm studies. The coefficient of determinant indicates the degree of fitting of the kinetic and isotherm with experimental data and it is given by Eq. (12).

12 $${R}^{2}=\frac{{\sum \left(Qecal-Qmexpt\right)}^{2}}{{\sum \left(Qecal-Qmexpt\right)}^{2}+{\left(Qcal-Qmexpt\right)}^{2}}$$

where Qexpt represents the amount of adsorbate adsorbed by the adsorbent during the experiment in mg/g, Qcal is the amount of adsorbate obtained by kinetic and isotherm modelling in mg/g, and Qmexpt stands for the average of Qexpt in mg/g. Reduced chi square is used to indicate the discrepancy between the experimental and calculated data, which is used to judge the goodness of fitting for experimentally obtained data. Reduced chi square is chi square per variance and is given by Eq. (13).

13 $$\frac{\sum_{i=1}^{n}\frac{{\left(Qecal-Qemeas\right)}^{2}}{Qemeas}}{v}$$

where Qecal is the calculated adsorption capacity, Qemeasured represents the measured adsorption capacity, and v denotes the degree of freedom. Finally, Origin Pro software was used for data fitting and error analysis^57,58^.

### Adsorption thermodynamics

The adsorption thermodynamics is of great importance since it provides information about the spontaneous or non-spontaneous nature of the adsorption process. Moreover, it helps to determine whether the process is endothermic or exothermic, the energy associated with the adsorption process, and the feasibility of the adsorption. The thermodynamic study of the adsorption of malachite green dye from an aqueous solution was conducted by varying the temperature of the system (25, 35, 45, 55, and 65) while holding other variables constant at their optimum values (pH of 6, contact time of 40 min, adsorbent dosage of 0.1 g/100 mL, and dye concentration of 100 mg/L)^51^. The Van't Hoff equation was used to determine the three essential parameters, namely enthalpy change (ΔH^o^), entropy change (S^o^) and Gibbs free energy (ΔG^o^) by employing Eqs. (13)–(16).

14 $$ \Delta G = - RT \,ln \, KC $$

15 $$K\mathrm{C }=\frac{qe}{Ce}$$

16 $$ {{ln}} \,\, KC = \frac{\Delta S}{R} - \frac{\Delta H}{{RT}} $$

17 $$ \Delta G = \Delta H - T\Delta S $$

where, R is the universal gas constant (8.314 J/mol. K), KC represents the thermodynamic constant, and T is the absolute temperature (K). Ce denotes the amount of MB dye concentration at equilibrium; qe represents the amount of dye adsorbed on the adsorbent at equilibrium (mg/g). The changes in entropy, enthalpy, and Gibbs free energy are denoted by the letters $\Delta$G, $\Delta$S, and $\Delta$H, respectively. The slope and intercept from the plot between ln KC and 1/T were used to calculate the values of $\Delta$H and $\Delta$S ^59^.

### Reusability and regeneration study

Industries are still facing the problem of solid waste disposal, including spent adsorbents^60^. Regeneration is a one-of-a-kind method that allows exhausted adsorbents to regain their adsorption ability by desorbing pre-adsorbed impurities^61^. Regeneration of spent adsorbent is required to eliminate solid hazardous waste for sustainable development^60^. It is frequently thought of as a less expensive and superior alternative to replacing the adsorbents. The regeneration process is influenced by the experimental temperature, pH, time, and processing^61^. The regeneration of the used *Rumex abyssinicus*-derived activated carbon was carried out using chemical methods and then used for several cycles to examine the reusability potential of the prepared adsorbent. The methods used by^43,45^ were followed for the regeneration of the used adsorbent.

### Ethical approval and consent to participate

All procedures are carried out per the institution's policies and rules. The institution's ethical clearance committee gave its approval to each experimental protocol. During sample collection, our institute gave a support letter to permit sample collection, and Addis Ababa Science Technology University permitted sample collection.

## Result and discussion

### Adsorbent characteristics

**Figure 1:**
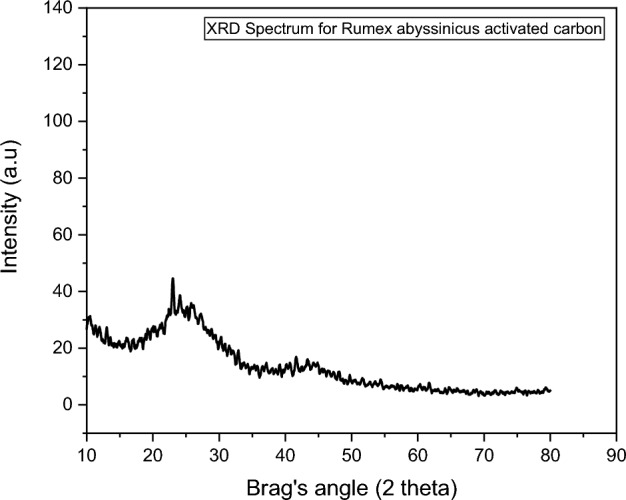
Crystalline structure analysis for *Rumex abyssinicus* based activated carbon.

The crystalline nature of the activated carbon synthesized from *Rumex abyssinicus* was examined using XRD, and the result of the finding is depicted in Fig. 1. From the XRD analysis, it can be observed that the general structure of the adsorbent is amorphous. Basically, amorphous surfaces are suitable for the adsorption of multipollutant varying in size and structure compared to crystalline structures. ^62^.

The BET-specific surface area of an activated carbon synthesized from *Rumex abyssinicus* was determined by employing the principle of nitrogen gas adsorption-desorption^63^. During BET-specific surface area analysis of the adsorbent under consideration, a degassing temperature and time of 200 °C and 1 h were guaranteed, respectively. Fundamentally, the higher the specific surface of the adsorbent, the greater the affinity for pollutant adsorption will be due to the availability of sufficient active sites at the specified surface of the adsorbent^64^. Normally, the rate of adsorption is highly dependent on the nature of the adsorbent material and its surface area. Owing to their more adsorbing sites, materials with a higher surface area increase the extent of the adsorption. On the other hand, porous materials provide a larger surface area, leading to both higher removal efficiency and adsorption capacity. Fundamentally, finely dividing (powdering) the adsorbent increases the surface area of the adsorbent, which eventually enhances the adsorption performance. The specific surface area of the activated carbon (*Rumex abyssinicus*) was found to be 962.3 m^2^/g. This extremely high specific surface area enables the adsorbent in minute quantities to adsorb substantial pollutants. Compared to biomass-based activated carbons, the specific surface area recorded in this study is higher. For instance, water hyacinth-derived activated carbon^65^, Rumex-abyssinicus based activated carbon 524 m^2^/g ^43^, Parthenium hysterophorus based activated carbon 265 m^2^/g ^66^.

**Figure 2:**
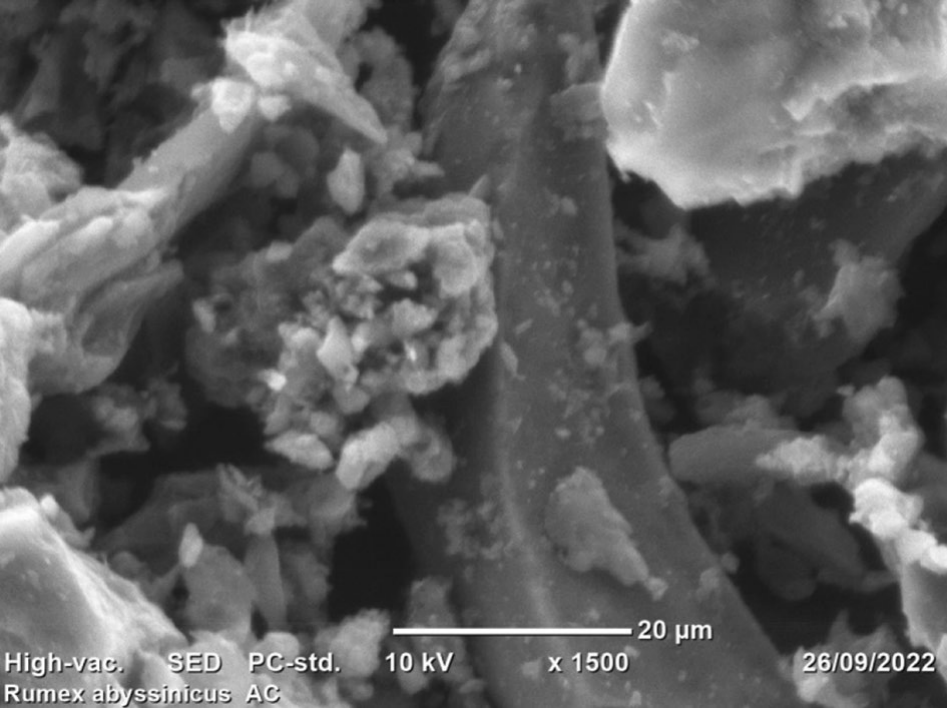
SEM morphology of activated carbon synthesized from *Rumex abysiniccus*.

The morphological analysis of the prepared adsorbent was undertaken using SEM, and the findings are depicted in Fig. 2. It can be observed from the image that the adsorbent is determined to have a porous structure, which enables it to adsorb pollutants varying in size and structure^67^. Morphological cracks such as holes and irregular shapes are observed due to the chemical and thermal activation undertaken to enhance the adsorption capacity of the activated carbon^68^. Similar findings were reported by ^42,43^ for *Rumex abyssinicus*-derived activated carbon applied for dye decolorization.

**Figure 3:**
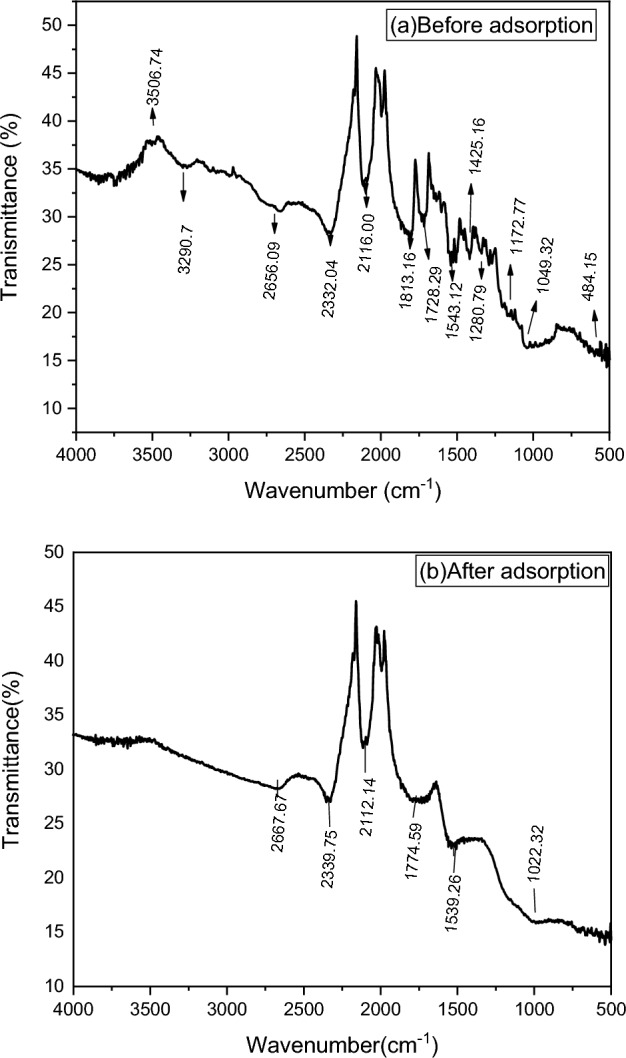
FTIR analysis of *Rumex abyssinicus*-derived activated carbon before (**a**) and after adsorption (**b**).

The functional group analysis of *Rumex abyssinicus*-based activated carbon before and after adsorption was successfully carried out using FTIR, as shown in Fig. 3a, b, respectively. The adsorbent was found to be composed of several functional groups capable of adsorbing various pollutants varying in structure and size^69^. For FTIR analysis of *Rumex abyssinicus* activated carbon before adsorption, the broad peaks observed at 3506.74 and 3290.70 cm^−1^ are attributed to –OH containing functional groups like hydroxyl and water. On the other hand, the shoulder peak observed at 2656.09 cm^−1^ demonstrates the vibration of carboxyl functional groups. The clear, sharp peaks observed between 1900 and 1700 cm^−1^ are attributed to the existence of C=O or C=C stretching vibrations of acid derivatives, which are characteristics of the carbonyl group stretching from aldehydes and ketones. A smaller peak located at 1543.12 cm^−1^ is associated with the stretching motion of aromatic rings. The relatively less intense peak observed in the region of 1500–1200 cm^−1^ corresponds to the stretching motions of -C-H groups. The wider peaks observed at 1172.27 and 1049.32 cm^−1^ are attributed to C-O bonds. Finally, the peak located at 484.15 cm^−1^ indicates the presence of H_3_PO_4_^47^. Compared to the FTIR spectra before adsorption, the pollutant-loaded activated carbon displayed significant changes in peaks. Diminishing and shifting peaks were observed after adsorption. The significant shifts (2656.09 cm^−1^ to 2667.07 cm^−1^), (2332.04 cm^−1^ to 2339.75 cm^−1^), (2116.00 cm^−1^ to 2112.94 cm^−1^), (1813.16 cm^−1^ to 1774.59 cm^−1^), (1543.12 cm^−1^ to 1539.36 cm^−1^), and (1049 cm^−1^ to 1022.32 cm^−1^) were observed from before adsorption to after adsorption, reflecting the effect of carboxyl, C=O, C=C, –C–H, and C–O, on the *Rumex abyssinicus*-based activated carbon surface during adsorption. Furthermore, the vanishing of several peaks such as 3506.74, 3290.70, and 1728.29, 1425.46, 1280.79, 1172.77, and 484.15 cm^−1^ were observed after adsorption, indicating no more presence of H_3_PO_4_ and –OH in the sample.

**Figure 4:**
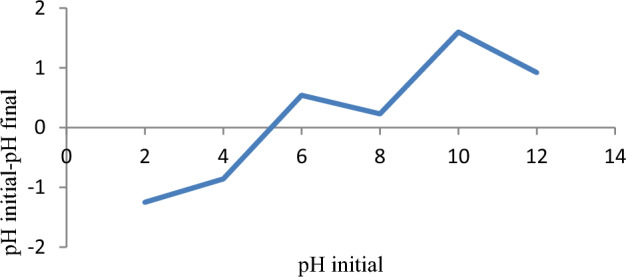
pHpzc of Rumex abyssinicus derived activated carbon.

The pH point of zero charge (pH at which the surface charge of an adsorbent becomes neutral) was determined to be 5.1, as shown in Fig. 4. A nearly similar finding, i.e., 5.04, was reported by^42^. Basically, at pHpzc the adsorbent has an equal amount of positively and negatively charged surfaces. More importantly, at the pH of the adsorbents below pHpzc, adsorption of anions favors due to the dominance of positive surface charges^70^. On the other hand, cations (positively charged substances like malachite green dye) are sufficiently adsorbed at a pH above pHpzc. Finally, the maximum removal of 99.9% attained at a pH of 6 supports the pHpzc concept.

### Batch adsorption performance

The adsorption of malachite green dye from an aqueous solution was performed in batch mode using activated carbon synthesized from the stem of the *Rumex abyssinicus* plant. The adsorption performance of the adsorbent material under varying experimental conditions is depicted in Table 2.

**Table 2:** Batch adsorption performances of *Rumex abyssinicus* for malachite green dye removal.

Run no.	pH	Dye con (mg/L)	Adsorbent dosage (g/100 mL)	Contact time (min)	Actual removal efficiency (%)
1.	9	50	0.050	60	85.67
2.	9	150	0.150	60	91.94
3.	6	150	0.150	60	89.35
4.	3	50	0.050	60	95.08
5.	6	150	0.1000	60	85.55
6.	6	50	0.050	20	84.26
7.	3	100	0.050	20	79.95
8.	6	150	0.150	20	76.14
9.	3	100	0.050	20	76.69
10.	6	150	0.100	20	66.66
11.	3	100	0.150	40	68.87
12.	9	50	0.150	40	95.41
13.	6	50	0.050	40	97.46
14.	3	100	0.050	40	88.88
15.	6	150	0.150	40	83.23
16.	9	50	0.150	60	94.28
17.	3	100	0.050	60	92.63
18.	9	150	0.150	60	90.95
19.	9	50	0.100	60	95.48
20.	9	150	0.050	60	71.28
21.	6	100	0.100	40	99.90
22.	3	50	0.050	40	98.14
23.	9	50	0.100	40	95.87
24.	6	50	0.150	40	86.78
25.	9	100	0.050	40	63.33
26.	3	100	0.150	20	62.40
27.	3	100	0.100	20	66.98
28.	6	100	0.100	20	89.95
29.	3	150	0.100	20	77.72
30.	9	150	0.100	20	71.62

The maximum and minimum removal efficiencies recorded in this study were found to be 99.9% and 62.4%, respectively. The maximum removal efficiency achieved in this study was recorded at optimum working conditions of pH 6, contact time of 40 min, initial dye concentration of 100 mg/L, and adsorbent dosage of 0.1 g/100 mL. On the other hand, the minimum removal efficiency (62.4%) was recorded at experimental conditions of pH, contact time, dye concentration, and adsorbent dosage of 3, 20 min, 100 mg/L, and 0.15 g/100 mL, respectively. Normally, various conditions should be considered while comparing the performances of different adsorbents on dye detoxification. These include the nature of the wastewater (real or aqueous), activation mechanism (thermal, chemical, or both), adsorbent dosage utilized, specific surface area, concentration of the pollutant, and so on. The adsorptive performance and surface area of *Rumex abyssinicus*-derived activated carbon applied for the removal of malachite green dye are compared with modified adsorbent materials such as SBA-15, MCM-41, and MCM-48. Consecutively, the surface area and maximum removal efficiency recorded in this study were found to be comparable. For instance^71^, reported a maximum removal efficiency of 99% for porous MCM-41 with a surface area of 1500 m^2^/g. Additionally, the 126 mg/g adsorption capacity of malachite green dye for MCM-41 has a surface area of 507 m^2^/g^72^, removal efficiency of 64% and surface area of 878 m^2^/g for MCM-41^73^, 97.1% removal efficiency for modified MCM-41^74^, surface area of 1420 m^2^/g was reported by^75^, 755.62 m^2^/g surface area and 140 61 mg/g adsorption capacity using SBA-15 ^76^. Furthermore, Table 3 presents a comprehensive comparison of previously done experiments on malachite green dye adsorption onto biomass-based adsorbents.

**Table 3:** Comparative study of biomass based activated carbons for malachite green dye adsorption.

Biomass	Removal efficiency/adsorption capacity	Isotherm model	Kinetics model	Thermodynamics	References
*Musa paradisiacal* L. leaf	> 99%	Redlich-Peterson	Avrami fractional model	Spontaneous and exothermic	^10^
*Syzyium samarangense*	95.5%	Langmuir	Pseudo second-order	Spontaneous and exothermic	^11^
*Salacca zalacca*	69.44 mg/g	Langmuir	Pseudo second order	–	^88^
Grape stalks	87.7%	Langmuir	Pseudo second order	Spontaneous and exothermic	^12^
Walnut shells	154.56 mg/g	Langmuir	Pseudo second order	Spontaneous and exothermic	^15^
*Bambusa tulda*	99.73%	Langmuir			^16^
*Salix alba* L	98.5%	Freundlich	Pseudo second order	Spontaneous and exothermic	^89^
*Cocos nucifera* shells	32.787 mg/g	Freundlich	Intraparticle diffusion		^17^
*Stipa tenacissima* L.) leaf powder	110.98 mg/g	Langmuir	Pseudo second order	Spontaneous and exothermic	^90^
Okra stalks waste	99.63 mg/g	Langmuir	Pseudo second order		^91^
Nutraceutical industrial fenugreek seed spent	105.00 mg/g	Langmuir	Pseudo second order	Spontaneous	^79^
*Catha edulis*	5.62 mg/g	Freundlich	Pseudo second order		^13^
*Lantana camara* L. stem	100 mg/g	Langmuir	Pseudo second order	Spontaneous and endothermic	^92^
*Rumex abyssinicus*	99.90%	Koble Corrigan	Pseudo second order	Spontaneous and endothermic	Present study

### Effects of operating parameters

**Figure 5:**
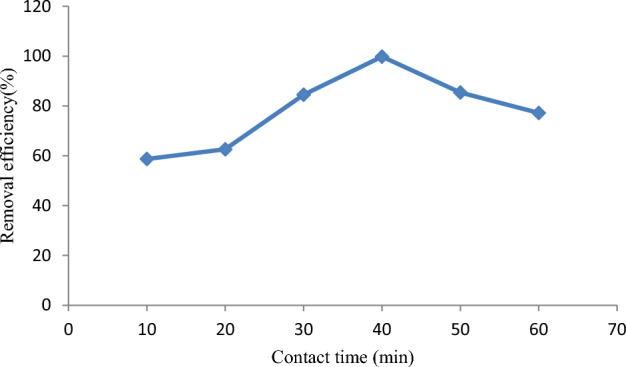
Effect of contact time on malachite green dye adsorption.

The effect of contact time on the removal of malachite green dye was examined on *Rumex abyssinicus* activated carbon for different contact times with a fixed adsorbent dose, pH, and initial concentration, as shown in Fig. 5. According to the results obtained, for a concentration of 100 mg/L, substantial malachite green dye removal occurred at 40 min, showing a 99.8% removal rate. The amount of malachite green dye adsorbed onto the adsorbent material increases with an increase in contact time; this is mainly due to the availability of a large number of vacant sites and pores on *Rumex abyssinicus* activated carbon adsorbent material for the adsorption of malachite green dye, and when time increases, the probability of dye molecules getting adsorbed increases. However, after 40 min of contact time, the removal efficiency decreased, showing the attainment of equilibrium. At 50 min, the removal efficiency reduced to 85.4%. This is probably due to the non-availability of the active site for malachite green dye ions to get adsorbed onto *Rumex abyssinicus*-derived adsorbent material and a decrease in the driving force^77^.

**Figure 6:**
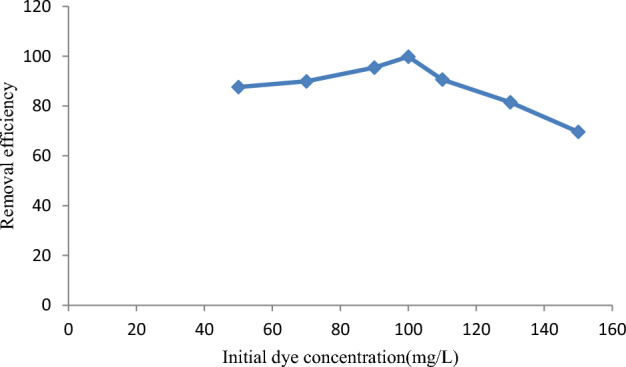
Effect of initial dye concentration on malachite green dye adsorption.

The initial malachite green dye concentration is an important factor in the adsorption capacity in terms of providing a higher driving force to overcome mass transfer resistances of the dye molecules between the aqueous solution and solid phases^78^. The effect of malachite green dye adsorption on various malachite green dye concentrations (50–150 mg/L) by activated carbon prepared from *Rumex abyssinicus* was thoroughly examined, as indicated in Fig. 6. It is observed that at 50 mg/L, the efficiency of adsorbed malachite green dye onto adsorbent material was 95.9%. However, increasing the malachite green dye concentration increased the removal, as it reached a maximum at a dye concentration of 100 mg/L. Accordingly, 100 mg/L was selected as the optimal initial malachite green dye adsorption for the coming experiments.

**Figure 7:**
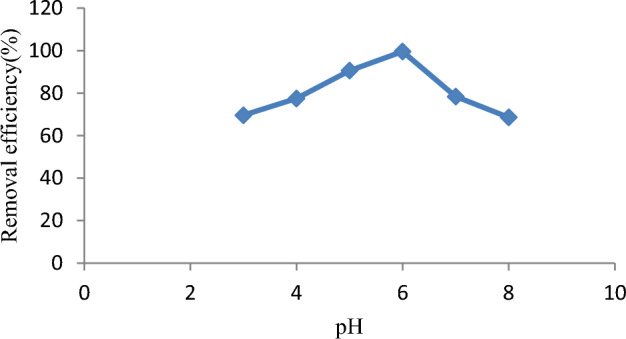
pH effect on malachite green dye adsorption.

The effect of pH on dye removal efficiency was studied by varying pH from 3 to 9, as depicted in Fig. 7. It was found that decreasing pH decreased the dye removal efficiency as the dye molecules competed with H^+^ for available active sites at lower pH values^79^. Normally, cationic dyes like malachite green dye are significantly adsorbed at higher pH values because of the absence of resistance^80^. Herein, the maximum removal efficiency of 99.9% was recorded at a pH of 6.

**Figure 8:**
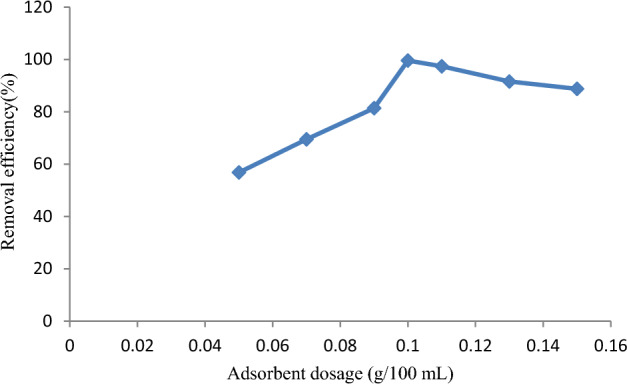
Adsorbent dosage effect on removal of malachite green dye.

Similarly, the effect of adsorbent dosage on dye removal efficiency was evaluated by varying the adsorbent dosage from 0.05 to 0.15 g/100 mL, as shown in Fig. 8. Initially, increasing the adsorbent dosage increased removal efficiency until 0.10 g/100 mL. However, a further increase in adsorbent dosage did not increase the removal efficiency because of the attainment of equilibrium.

### Adsorption isotherm

**Figure 9:**
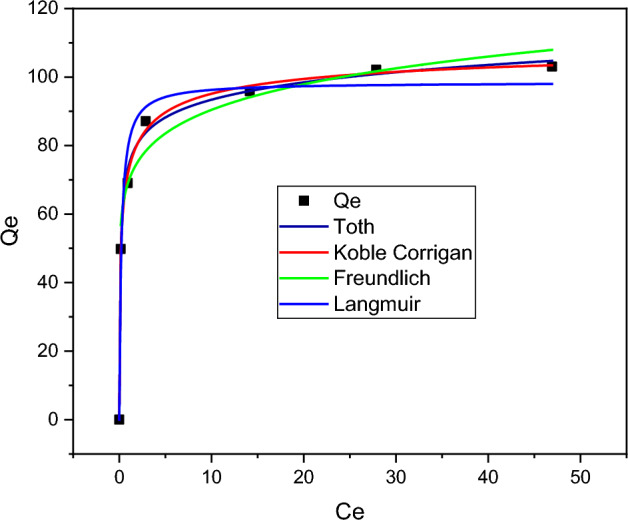
Adsorption isotherm model curve fitting.

Adsorption isotherms were assessed using the Langmuir, Freundlich, Toth, and Koble-Carrigan models, as shown in Fig. 9. The coefficient of determination (R^2^) and reduced chi-square were taken into consideration when selecting the best curve fit. As a result, it is evaluated that the Langmuir isotherm has a maximum R^2^ of 0.97 and a negligible reduced chi-square of 0.97008 compared to the Freundlich isotherm model. Furthermore, the maximum Langmuir adsorption capacity (Q_max_) and the Langmuir constant related to the free energy of adsorption (K_L_) were found to be 98.43 mg/g and 4.41 L/mg, respectively, demonstrating the effectiveness of the adsorption process. On the other hand, the Freundlich constant (K_F_) and an empirical parameter related to intensity (1/n) were determined to be 69.47 mg/g and 0.11, respectively, as given in Table 4. Additionally, the dimensionless separation factor constant (RL), which is used to estimate Langmuir’s isothermal feasibility, was found to be 0.0048. The applicability of three-parameter isotherm models for evaluating the nature of malachite green dye adsorption onto *Rumex abyssinicus*-based activated carbon was examined employing the Toth and Koble-Carrigan models. Basically, these three-parameter adsorption isotherm models are an amalgamation of Freundlich and Langmuir isotherm models, which further signify the homogeneity or heterogeneity of the adsorption sites. Accordingly, there is an insignificant discrepancy between Toth’s R^2^ (0.996) and Koble-Corrigan’s R^2^ (0.998). Moreover, the curve fitting analysis suggested that both Toth and Koble-Corrigan isotherms excellently fit the plot. On the other hand, the nk and nt values for the Koble-Corrigan, and Toth models are almost unity, indicating the homogeneity of the adsorption surfaces. It has been reported that the deviation of nk and nt values from unity describes the heterogeneity of the adsorption system, while n and t values being close to unity indicates monolayer adsorption^81,82^. Moreover, the maximum monolayer adsorption capacity predicted by Koble-Corrigan is very close to the one predicted by the Langmuir isotherm model, proving homogenous distribution sites. Comparatively, the best to poorest fitting regarding R^2^ can be ranked as Koble-Corrigan > Toth > Langmuir > Freundlich, while error analysis suggested a degree of fitness to be ranked as Koble-Corrigan > Toth > Freundlich > Langmuir. Generally, from the adsorption isotherm analysis result, it can be inferred that the adsorption process is favorable (0 < R_L_ < 1), normal (1/n < 1), homogenous and monolayer ^81,83^.

**Table 4:** Parameters for Langmuir and Freundlich isotherm models.

Isotherm model	Parameters
Langmuir	R^2^ = 0.97 K_L_ = 4.41 R_L_ = 0.0048 Q_max_ = 98.43 Reduced Chi-square = 0.97008
Freundlich	R^2^ = 0.93 K_F_ = 69.47 1/n = 0.11 n = 9.09 Reduced Chi-square = 0.037
Koble Corrigan	R^2^ = 0.998 A = 204.88 B = 1.83 nk = 0.9827 Reduced Chi-square = 0.0006
Toth	R^2^ = 0.996 Q_max_ = 97.44 nt = 0.991 K = 13.45 Reduced Chi Square = 0.015

### Adsorption kinetics

The adsorption kinetics model, which is used to determine the potential rate-determining step as well as has great significance in evaluating the time dependency of the dye adsorption, was determined by using PFO, PSO, intraparticle diffusion, and Boyd kinetics models. Accordingly, the PSO-kinetic model with an R^2^ of 0.99 and a reduced chi-square of 0.00023 was found to fit the data best. Moreover, there is a negligible discrepancy between Qe calculated (121.24 mg/g) and Qe experimental (120.94) for the PSO-kinetic model, as depicted in Table 5. The intercept of the intraparticle diffusion model (C) was not zero, suggesting that intraparticle diffusion does not exclusively limit the rates of adsorption. Hence, both film diffusion and bulk diffusion govern the adsorption process. Generally, the rate of malachite green dye adsorption onto *Rumex abyssinicus*-derived activated carbon is governed by chemical reaction, where both adsorbate and adsorbent control the rate of the adsorption process^34^.

**Table 5:** Kinetics parameters for malachite green dye adsorption.

Pseudo first order	Pseudo second order	Intraparticle diffusion	Boyd model
R^2^ = 0.91	R^2^ = 0.99	R^2^ = 0.94	R^2^ = 0.95
K_1_ (min^−1^) = 1.47	K_2_ (g/mg min) = 0.58	${k}_{d}$(mg/g min^0.5^) = 0.21	F = 0.92
Q_e_ cal (mg/g) = 104.51	Q_e_ cal (mg/g) = 121.24	C(mg/g) = 2.14	*B*_*t*_=2.028
Reduced Chi-square = 0.26	Reduced Chi-square = 0.004	Reduced Chi square = 0.0061	Reduced Chi square = 0.36

### Adsorption thermodynamics

**Figure 10:**
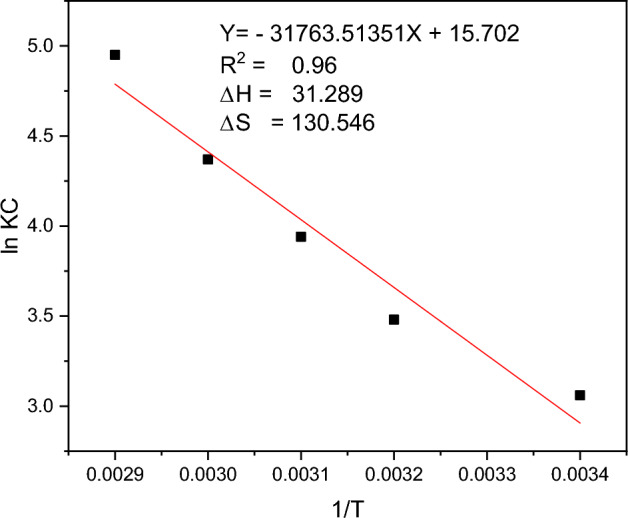
Adsorption thermodynamics for the adsorption of malachite green onto *Rumex abyssinicus* activated carbon.

The adsorption thermodynamics provide information about the energy associated with the adsorption being undertaken**.** Additionally, it helps to determine the nature of the adsorption (spontaneous or non-spontaneous). Basically, the adsorption process is said to be spontaneous if the Gibbs free energy of the system becomes negative and non-spontaneous if its Gibbs free energy is positive. In this regard, the *Rumex abyssinicus*-derived activated carbon applied in the removal of malachite green dye from aqueous solution was found to be thermodynamically spontaneous ($\Delta G<0$) and endothermic ($\Delta H= 31.289 \mathrm{kJ}/\mathrm{mol}$), as shown in Fig. 10. In general, from a thermodynamic perspective, the adsorption of malachite green dye onto *Rumex abyssinicus*-derived activated carbon is feasible^84^.

### Reusability and regeneration studies

**Figure 11:**
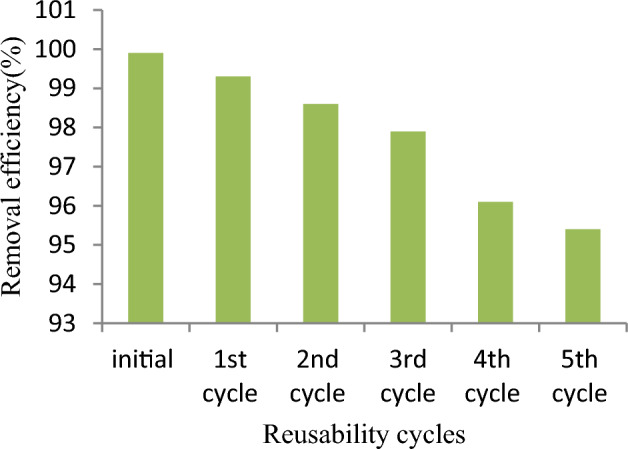
Reusability potential of *Rumex abyssinicus* derived activated carbon.

The chemically regenerated adsorbent was examined for reusability potential for five consecutive cycles, and the adsorption performance is shown in Fig. 11. As can be observed from the figure, the removal efficiency of malachite green dye onto reused *Rumex abyssinicus*-derived activated carbon ranged from 99.9 to 95.2%, demonstrating strong reusability potential. Normally, after repeated cycles of reusability, the performances of the activated carbon for pollutant detoxification declines gradually due to incomplete desorption and the decline of active sites found on the surface of the adsorbent.

### Adsorption mechanisms

In this specific study, the adsorption of malachite green is a surface phenomenon, which is a spontaneous process that facilitates adhesion of malachite green molecules from wastewater onto a surface of *Rumex abyssinicus* derived activated carbon adsorbent creating a film of the adsorbate on the surface of the adsorbent. The adsorption mechanism covers various steps including molecular interactions, diffusion of dye molecules through the boundary layer followed by intraparticle diffusion into the interior of the sorbent, either by monolayer or multilayer and finally adsorption on the surface of adsorbent^85^.The first step of the adsorption process is the migration or diffusion of impurities present in water into porous cavities within carbon particles. Once inside the pore, the impurity molecules are held by internal pore surfaces by weak electrostatic forces known as Vander Walls force^86,87^. Adsorption continues with the removal of solutes from solution until the amount of solute remaining in solution is in equilibrium with that at the surface. The mechanism adsorption on the surface of activated carbon is commonly due to its micropore present or the weak vander waals forces which can attract the impurities and is the result of specific interactions between functional groups via formation of donor–acceptor complexes^86,87^. On the other hand, the shifting of FTIR bands (2656.09 to 2667.07), (2332.04 to 2339.75), (2116.00 to 2112.94), (1813.16 to 1774.59), (1543.12 to 1539.36), (1049 to 1022.32) before and after adsorption reflects that carboxyl, C=O, C=C, –C–H, C–O played an important role in the adsorption process. Finally, the hydrophobicity of the impurity molecule and the affinity of the contaminant molecule towards carbon or both facilitate the adsorption of contaminants from waste water^75^.

## Conclusion

The Rumex-abyssinicus-derived activated carbon, which was synthesized through chemical and thermal activation, was characterized for pHpzc, SEM, FTIR, BET, and XRD. The result of the analysis has shown a pHpzc of 5.1 and an extremely high BET-specific surface of 962.3 m^2^/g. On the other hand, the presence of multiple functional groups like –OH (3290.70 cm^−1^), carboxyl (2656.09 cm^−1^), C=O or C=C (1900–1700 cm^−1^), stretching motion of aromatic rings (1543.12 cm^−1^), stretching motion of –C–H (1500–1200 cm^−1^) and C–O (1172.27 and 1049.32 cm^−1^) were identified by FTIR. Additionally, XRD and SEM analysis revealed the amorphous nature of the adsorbent with a heterogeneous surface. The removal efficiency recorded throughout the study ranged from 99.9 to 62.4%. The maximum removal efficiency was recorded at optimum conditions of pH 6, a contact time of 40 min, an adsorbent dosage of 0.1 g/100 mL, and a dye concentration of 100 mg/L. The effects of experimental variables such as pH, contact time, adsorbent dosage, and initial dye concentration were thoroughly examined. The adsorption isotherm, kinetics, and thermodynamics studies were undertaken with the aim of determining the nature of the adsorption, mechanism, and heat transfer associated with the adsorption process. Accordingly, the Koble-Corrigan isotherm and PSO kinetics models were found to be descriptive, with maximum R^2^ values of 0.998 and 0.99, respectively. This showed that the adsorption process was chemosorption with a homogenous surface, in which the surface of the adsorbent was saturated with a single attachment of pollutants. The thermodynamics study revealed the nature of adsorption to be spontaneous and endothermic. The regeneration of the used adsorbent was successfully undertaken using a chemical method that was later applied for the adsorption of malachite green dye for five cycles. The reusability study demonstrates the excellent adsorption capability of the *Rumex abyssinicus*-derived activated carbon, with removal efficiencies ranging from 99.9 to 95.2%. Finally, this research showed that *Rumex abyssinicus*-derived activated carbon can be taken as a potential candidate to be used for the decolorization of textile effluents. However, it is recommended to functionalize the surface of the adsorbent for enhanced adsorption capability and to study the column analysis.

## Data Availability

All data are included in the manuscript.
